# The Evaluation of the Oxidative Stress Parameters in Patients with Primary Angle-Closure Glaucoma

**DOI:** 10.1371/journal.pone.0027218

**Published:** 2011-11-11

**Authors:** Dong Chang, Qian Sha, Xuefei Zhang, Peipei Liu, Shengzhong Rong, Tao Han, Ping Liu, Hongzhi Pan

**Affiliations:** 1 First Affiliated Hospital, Harbin Medical University, Harbin, China; 2 Eye Hospital of Heilongjiang Province, First Affiliated Hospital, Harbin Medical University, Harbin, China; 3 Second Affiliated Hospital, Harbin Medical University, Harbin, China; 4 Public Health School, Harbin Medical University, Harbin, China; University of Saarland Medical School, Germany

## Abstract

**Objective:**

To clarify the presence of oxidative stress in patients with primary angle-closure glaucoma (PACG) and to investigate the relationship between oxidative stress and PACG.

**Methods:**

Fifty patients with primary angle-closure glaucoma and fifty healthy controls of matched age and gender were included in the study prospectively. Serum samples were obtained to detect the oxidation degradation products malondialdehyde (MDA), conjugated diene (CD), 4-hydroxynonenal (4-HNE), advanced oxidation protein products (AOPP), protein carbonyl (PC), ischemia-modified albumin (IMA) and 8-hydroxydeoxyguanosin (8-OHdG).

**Results:**

The concentration of MDA and CD in PACG patients was significantly higher than those of the control subjects (*P*<0.05, *P*<0.01). The serum 4-HNE concentrations were increased in PACG patients, but the differences with those of the healthy controls were not statistically significant. Compared to normal subjects, there was significant higher in serum AOPP and PC in PACG patients (*P*<0.01). PACG patients had higher levels of 8-OHdG in serum with respect to the comparative group of normal subjects (*P*<0.01). When plasma IMA levels in the PACG group were compared with those in the control group, significant increases in IMA were observed in the former (*P*<0.05).

**Conclusions:**

Our study demonstrated that IMA is a new biomarker available for assessing oxidative stress in PCAG. Oxidative stress is an important risk factor in the development of primary angle-closure glaucoma. Increased levels of oxidative stress products may be associated with primary angle-closure glaucoma.

## Introduction

Glaucoma is a progressive optic neuropathy and is the leading cause of blindness in the developing and industrialized countries. Although the initiating causes leading to glaucoma are unknown, oxidative and nitrative stress appears to play a role in the progressive neuronal death that is characteristic of glaucomatous optic nerve damage [1]–[3]. Increased markers of oxidative stress that have been reported in glaucoma include protein nitrotyrosine, carbonyls in proteins, lipid oxidation products and oxidized DNA bases [4]–[6]. It is concluded that oxidative stress has a pathogenic role in glaucoma.

The pathogenesis of glaucoma is multifactorial, and the precise mechanisms are unclear. Various factors may play an elementary role in the pathologic course of glaucoma, such as genetic factors, increased levels of glutamate, changes in nitric oxide metabolism, and vascular changes [7]–[9]. One factor, which is increasingly important in the pathogenesis of glaucoma, is oxidative stress. A number of studies in vitro and in vivo suggested that oxidative stress is increased in glaucoma patients. Abu-Amero et al reported in patients with glaucoma demonstrated the occurrence of mutations in the mitochondrial genome and a reduced mitochondrial respiratory activity in comparison to control subjects [10]. Moreover, it has been ascertained that the antioxidative capacity in the aqueous humor of patients with glaucoma is markedly reduced compared to nonglaucomatous eyes [2].

The relationship between oxidative stress and primary open-angle glaucoma (POAG) has already been reported [2]–[6], [11]–[13]. Sacca et al reported that a statistically significant correlation was found among human trabecular meshwork DNA damage, visual field damage, and intraocular pressure, and concluded that oxidative stress could induce human trabecular meshwork degeneration, resulting to an intraocular pressure increase, thus priming the glaucoma pathogenetic cascade [4]. Feilchenfeld et al reported that increased nitrotyrosine (a footprint for oxidative injury) is present in blood vessels and astrocytes in the pre-laminar optic nerve head in human primary open-angle glaucoma than controls [5]. However, the relationship of oxidative stress and primary angle-closure glaucoma (PACG) is unknown. It is presumed that oxidative stress also play an important role in the development of primary angle-closure glaucoma.

Taking these studies into account, this study is to evaluate the oxidative stress parameters in PACG patients and investigate the relationship between oxidative stress and PACG.

## Results

The clinical characteristics of the PACG patients and control subjects are shown in Table 1. Age and gender of the patient were not significantly different from those of the control.

**Table 1:** Clinical characteristics of PACG patients and health control.

	Health group	glaucoma patients
**N**	50	50
**Age (years)**	60.94±6.95	59.04±8.42
**Sex (F/M)**	25/25	25/25
**IOP left eye (mmHg)**	16.0±2.4	27.8±8.7*
**IOP right eye (mmHg)**	15.8±2.3	24.3±5.5*
**Cholesterol (mmol/L)**	4.99±0.65	5.04±0.71
**Triglycerides (mmol/L)**	1.10±0.37	1.18±0.40
**Glucose (mmol/L)**	4.57±0.72	4.67±0.51
**Systolic blood pressure(mmHg)**	127.2±5.8	124.9±8.9
**Diastolic blood pressure(mmHg)**	79.6±4.4	78.0±6.9

*Data are means±SD.*

*P<0.01 vs healthy subjects.

As shown in table 2, MDA significantly increased in patients' serum compared with normal subjects (*P*<0.01). Conjugated dienes significantly increased in patients compared to the controls (P<0.05). The concentration of 4-HNE was a bit higher in PACG patients, but there was no great difference between the glaucoma group and the healthy control.

**Table 2:** Serum levels of MDA, conjugated dienes and 4-HNE.

Group	MDA (nmol/ml)	conjugated dienes (ABSU)	4-HNE(nmol/ml)
**Health control**	3.51±0.84	0.350±0.107	14.16±2.98
**Glaucoma patients**	4.35±0.81**	0.404±0.102*	15.25±3.28

*Data are means±SD.,*

*P<0.05,

**P<0.01 vs healthy subjects.

Data presented in Table 3 show that the levels of AOPP, serum protein carbonyl and serum 8-OHdG significantly increased in patients compared with the control (*P*<0.01). And the concentration of serum IMA greatly increased in patients compared with normal subjects (*P*<0.05).

**Table 3:** Serum levels of AOPP, protein carbonyl and 8-OHdG.

Group	AOPP (µmol/L)	Protein carbonyl (nmol/ml)	8-OhdG (nmol/ml)	IMA (ABSU)
**Health control**	15.96±3.51	2.94±0.72	22.44±4.95	0.409±0.036
**Glaucoma patients**	17.88±3.42**	3.42±0.76**	25.18±5.33**	0.423±0.030*

*Data are means±SD.,*

*P<0.05,

**P<0.01 vs healthy subjects.

## Discussion

Glaucoma is the second leading cause of blindness after cataract in the world. Glaucoma is a heterogeneous group of eye conditions with manifestation as early as birth to very late age of onset and are among most common cause of blindness worldwide. Recent report estimates that there will be 60.5 million people with primary glaucoma in 2010 and 79.6 million by 2020 resulting in bilateral blindness in 8.4 and 11.2 million people by the corresponding years, respectively [14]. In general, glaucoma is broadly classified into three major groups: (i)primary open angle glaucoma (POAG); (ii) primary angle-closure glaucoma (PACG); and (iii) primary congenital glaucoma [15], [16]. Among those subtypes PACG is the most common form of the glaucoma in China.

The common denominator of these diseases is the involvement of free radicals. Numerous scientific investigations have confirmed the presence of oxidative stress in ocular diseases. Indeed, reactive oxygen species (ROS) may play a significant role for the pathophysiology in glaucoma [2], [4], [17]. In ophthalmology, oxidative stress has been reported to induce and facilitate progression of cataracts and diabetic retinopathy [18], [19]. In glaucoma, antioxidant levels decrease in the aqueous humor in patients with POAG compared with in the aqueous humor of patients with cataracts, suggesting that peroxidation may be involved in the development of glaucoma [2].

Oxidative stress plays a critical role in the development and progression of glaucoma, but the mechanism is not clear. The possible sources of increased oxidative stress might include increased generation of free radicals or impaired anti-oxidant defence system. In general, oxidative stress, including ROS, can cause oxidative damage to DNA, proteins, and lipids, leading to DNA and protein modification and lipid peroxidation, and many clinical conditions are associated with increased indices of oxidant stress.

Recent datas indicate that the oxidative stress plays an important role in the pathogenesis of glaucoma. POAG may be associated with increased lipid peroxidation and oxidative DNA damage caused by oxidative stress. Increasing evidences in both experimental and clinical studies suggests that there is a close link between oxidative stress and glaucoma [1], [2], [20]. It had been considered that oxidative stress contribute to the pathological processes of glaucoma [6].

Oxidatants are highly reactive compounds with a half-life of only seconds. It is generally not feasible for in vivo determination. In contrast, lipids, proteins, carbohydrates and DNA, after being modified by oxy-radicals, have lifetimes ranging from hours to weeks, can be measured with biochemical assays, which makes them ideal markers of oxidative stress. Many biomarkers have been developed to evaluate oxidative stress. These markers include lipid peroxidation products (such as acrolein, MDA, conjugated dienes, F_2_-isoprostanes and 4-hydroxynonenal), protein oxidation products (such as AOPP, protein carbonyl) and DNA damage products (8-OHdG).

The resultant end products, MDA, F_2_-isoprostanes, CD or 4-hydroxynonenal (HNE) are well-known markers in the pathologic molecular process in oxidative stress. In this study, we selected three biomarkers of lipid peroxidation that are widely used, sensitive, and appropriate for use in large studies, CD, MDA and 4-HNE. CD is the initial formation of a lipid peroxide. MDA is a decomposition product of peroxidized polyunsaturated fatty acids. 4-HNE derives from ω-6 polyunsaturated fatty acids like linoleic and arachidonic acid whose conjugated double bonds are an easy target for species that can extract a hydrogen atom or add to a double bond [21].

Production of ROS and lipid peroxidation are increased in glaucoma patients. Babizhayev and Bunin [22] reported increased levels of lipid peroxides in the aqueous humor, trabecular meshwork, and Schlemm's canal in POAG compared with control eyes, and suggested that lipid peroxidation was responsible for destruction of the trabecular meshwork and Schlemm's canal. In addition, Bunin et al [23] reported that destruction of the drainage system in POAG is accelerated by lipid peroxidation. Lipid peroxidation products have been found in significantly higher concentrations in the aqueous humour and trabecular tissue of glaucoma patients compared with control subjects [22]. Increased vitreous and retina MDA levels were also detected in rats with elevated intraocular pressure (IOP) [24], [25]. In our study, we found that PACG patients had significantly higher MDA and conjugated dienes compared with control subjects. Therefore, the concentration of 4-HNE in patients with PACG was elevated in comparison to control, but the differences with those of the healthy controls were not statistically significant.

AOPP, which formed during oxidative stress by the action of chlorinated oxidants, mainly hypochlorous acid and chloramines (produced by myeloperoxidase in activated neutrophils), has begun to attract the attention of various investigators [26], [27]. AOPP was described by Witko-Sarsat et al^15^for the first time. They are elevated in patients with renal insufficiency and diabetes mellitus [28], [29]. In our study, we have also determined the level of CD, AOPP and protein carbonyl, three products protein oxidation, which were increased, and demonstrated that there were protein oxidative damage in PACG. Additional, the results show that AOPP is a biomarker available for assessing oxidative stress in PCAG, too.

Biomarkers of protein oxidation are often applied when a battery of markers of oxidative stress status is being studied. Protein carbonyl formation is a widely utilized marker for protein oxidation [30]. Protein carbonyl (PC) content is the most general and well-used biomarker of severe oxidative protein damage. The measurement of carbonyl groups is considered to be a good estimation for the extent of oxidative damage to proteins. Serum protein carbonyl levels revealed a significant increase in glaucoma patients compare with healthy controls [31]. Protein carbonyl formation was also identified via proteomic analysis in a chronic pressure induced rat model of glaucoma [20]. In this study, we found that the level of protein carbonyl in PACG was increased significantly. The effect of carbonyl formation on the function of glutamine synthetase remains to be elucidated but may have potential consequences on retinal ganglion cell death associated with glaucoma.

Oxidative deoxyribonucleic acid (DNA) damage can lead to DNA protein cross linking, strand breaks and base modifications. Oxidative DNA damage is assessed by measuring levels of 8-OHdG, an indicator of oxidative DNA damage. It has been regarded as a novel biomarker of oxidative DNA damage in vivo. Elevated intraocular pressure due to reduction in aqueous outflow facility is a major causal effect in glaucoma [32]. The eye's outflow system consists of a series of endothelial cell-lined structures in the angle of the anterior chamber, which also include the trabecular meshwork. Therefore, the contents of 8-OHdG in serum could be act as a sensitive biomarker for the glaucoma patients. Levels of 8-OHdG was determined in human trabecular meshwork (HTM) specimens collected from patients with primary open-angle glaucoma (POAG) [4]. The relationship between DNA oxidation, IOP and visual field (VF) damage and disease duration were also evaluated. A statistically significant correlation was also found among oxidative DNA levels, visual field damage and intraocular pressure. In our study, we found that PACG patients had high 8-OHdG concentrations. It was concluded that oxidative DNA damage may induce human trabecular meshwork degeneration, leading to increase IOP.

Ischemia-modified albumin is a new biochemical marker. It has been recently reported that IMA is a highly sensitive biomarker, reflecting the myocardial ischemic condition prior to progression to myocardial necrosis [33]–[35]. During acute ischemic conditions, the metal binding capacity of albumin to transition metals such as copper, nickel, and cobalt is reduced, generating a metabolic variant of the protein. This change is quantifiable and commonly known as IMA [36]. IMA has been studied primarily in selected populations thought to display myocardial involvement only in the absence of confounding clinical conditions. However, other organs seem to be responsible for the increase in IMA. High IMA concentrations do not seem to depend purely on myocardial involvement. IMA may not be specific for cardiac ischemia. There are several data on IMA in patients with different states with ischemia of non-cardiac origin such as systemic sclerosis, peripheral vascular disease, skeletal muscle ischemia and diabetes mellitus [37]–[39]. But no one concerns PACG.

Ischemia plays a role in the pathogenesis of several diseases [40], [41]. It is believed that ischemia-induced injury plays an important role in some retinal diseases such as glaucoma, including open angle glaucoma, closed angle glaucoma, and normal-tension glaucoma, and central retinal vessel occlusions. IMA, a metabolic variant of albumin, emerges in ischemic states. There is as yet no definitive conclusion as to how IMA emerges. Elevated IMA levels may result from increased oxidative stress, caused by ischemia reperfusion injury or other mechanisms linked to primary reduction of coronary blood flow or muscle damage [42], [43]. Some evidence suggest that IMA levels increase in various acute ischemic conditions, such as myocardial ischemia, skeletal ischemia, cerebral ischemia, mesenteric ischemia, and pulmonary ischemia [44], [45]. But there is no study in the literature evaluating the IMA levels in patients with PACG. In our study, we observed that IMA levels were higher in PACG patients compared with that in the healthy control subjects. This is the first time to our knowledge in the literature that the correlation between PACG and serum IMA levels has been compared using a healthy control group. Moreover, our study demonstrated that IMA is a new biomarker available for assessing oxidative stress in PCAG.

In conclusion, we found that there were severe lipid peroxidation, oxidative DNA damage and protein damage in the patients with PACG. These findings suggested the possibility that increased oxidative stress may be associated with in PACG. Therefore, assessment of oxidative stress in PCAG patients may be important for the therapy and prevention of glaucoma.

## Materials and Methods

### Subjects

The studied group consisted of 50 patients with PACG, who were recruited from the First Affiliated Hospital of the Harbin Medical University and the Eye Hospital of Heilongjiang Province. All subjects underwent a complete ophthalmic examination including best corrected visual acuity measurements, slit-lamp biomicroscopy, Goldmann applanation tonometry, gonioscopy, fundoscopy. The diagnostic criteria for PACG were as follows: open and non-occludable anterior chamber angles with gonioscopy (Volk 3 Mirror Gonio Lens, Mentor, USA), glaucomatous optic disc cupping was identified as a vertical cup-to-disc ratio of optic nerve head 0.6 or more, difference of the vertical cup-to-disc ratio 0.2 or more between both eyes, rim width at superior portion (11–1 h) or inferior portion (5–7 h) of 0.2 or less of disc diameter or the presence of nerve fiber layer defect. The exclusion criteria of this study were as follows. Subjects with other ophthalmic conditions such as primary open-angle glaucoma, pigment dispersion glaucoma, exfoliative glaucoma, trauma, any other type of secondary glaucoma were excluded. Subjects who took vitamins, steroids, or medications were excluded. Active smokers and subjects with smoking were also excluded. Subjects who had systemic diseases such as diabetes mellitus or hypertension were excluded.

Fifty healthy, age-matched subjects were also included for the control. The control subjects were recruited from subjects who came to the same hospital for an annual refractive check-up. The control subjects did not have any history of ocular diseases, and underwent the same examinations as the patients by the same investigator. None of the control subjects was taking any medication. Active smokers and subjects who had smoked were also excluded. All subjects gave written informed consent to participate in this study.

### Blood sampling

Venous blood samples were collected after 12 h overnight fasting from each subject. The samples were placed on ice and centrifuged with an hour at 3500 rpm, 4°C for 15 minutes, and the supernatants were stored at −20°C and determination of the samples occurred within 3 months.

### Laboratory Analysis

#### Malondialdehyde assay

Malondialdehyde (MDA) was measured as thiobarbituric acid-reacting substance (TBARS) production in the following manner. 0.1 ml of sample was added to a 1∶1∶1 (vol/vol/vol) solution of trichloroacetic acid (15%, wt/vol), thiobarbituric acid (0.375%, wt/vol), and hydrochloric acid (0.25 M). The mixture was heated at 100°C for 30 min. The mixture was immediately cooled and then centrifuged (3,500 g for 5 min) to remove undissolved materials. Then the absorbance at 532 nm was determined. The amount of TBARS was calculated from comparison with authentic malondialdehyde.

#### Conjugated dienes assay

Conjugated diene (CD) determined according to the method of Ward *et al* [46]. Conjugated dienes extracted from plasma using a 2∶1 (vol/vol) mixture of chloroform and methanol. 4 ml of the chloroform-methanol mixture, preheated to 45°C, were added to 0.1 ml of serum. The mixture was then vigorously mixed (with a vortex machine) for 2 min, then mixed with 2.0 ml of distilled water acidified with 0.1 M HCl to a pH of 2.5. After agitation that used a vortex instrument, the material was subjected to centrifugation (2,000 g for 5 min), and 1.5 ml of the lower layer was aspirated, transferred to a test tube, and dried under a flow of nitrogen gas. The residue was reconstituted with 1.0 ml of heptane and measured spectrophotometrically at 233 nm. The results were reported as absorbance units (ABSU).

#### Advanced oxidation protein products assay

Advanced oxidation protein products (AOPP) were quantified as described of Witko-Sarsat V [47]. We placed 200 µl of serum diluted 1∶5 in phosphate-buffered saline into each well of a 96-well microtitre plate and added 20 µl of acetic acid to each well. For the standards, we added 10 µl of 1.16 M potassium iodide (Sigma, St Louis, MO, USA) to 200 µl of chloramine-T solution (0 to 100 µmol/L) (Sigma, St Louis, MO, USA) in a well and then added 20 µl of acetic acid. The absorbance of the reaction mixture was immediately read at 340 nm against a blank consisting of 200 µl of phosphate-buffered saline, 10 µl of 1.16 M potassium iodide, and 20 µl of acetic acid. AOPP concentrations are expressed as µmol/L of chloramine-T equivalents.

#### Protein carbonyl assay

Protein carbonyl concentrations in plasma were measured by the spectrophotometric assay described by Reznick and Packer [48]. Briefly, to 200 µl plasma, 4 ml of 10 mmol 2, 4-dinitrophenylhydrazine (DNPH) in 2 mol/L HCl was added. In another tube, 4 ml of 2 mol/L HCl was added to 200 µl plasma (of the same patient). The tubes were left in the dark for 1 h at room temperature and were mixed by vortex every 15 min. Five milliliters of 20% trichloroacetic acid solution was then added to both tubes for a 10 min incubation on ice, after which the tubes were centrifuged (3000× *g*, 5 min, 4°C). The supernatant fluid was discarded and another wash was performed by using 4 ml 10% trichloroacetic acid. The protein pellets were broken mechanically and washed 3 times with ethanol–ethyl acetate to remove free DNPH and lipid contaminants. The final precipitates were dissolved in 2 ml of 6 mol guanidine hydrochloride/L, and insoluble materials were removed by additional centrifugation (3000×*g*, 5 min, 4°C). The absorbance at λ = 370 nm was measured spectrophotometrically. The carbonyl content (nmol/ml) was calculated using ε_M_ = 22,000.

#### Ischemia-modified albumin assay

Reduced cobalt-toalbumin binding capacity [Ischemia-modified albumin (IMA) level] was analyzed using the rapid and colorimetric method described by Bar-Or et al [49]. Two hundred microliters of patient serum was placed into glass tubes and 50 ml of 0.1% cobalt chloride (CoCl_2_•6H_2_O; Sigma, Sigma-Aldrich Corporation, St. Louis, MO) in H_2_O was added. After gentle shaking, the solution was left for 10 minutes to ensure sufficient cobalt–albumin binding. Fifty microliters of dithiothreitol (DTT) (1.5 mg/ml H_2_O; Sigma) was added as a colorizing agent, and the reaction was quenched 2 minutes later by adding 1.0 ml of 0.9% NaCl. Specimen absorbencies were analyzed at 470 nm using a spectrophotometer. IMA was calculated from the difference between samples measured with and without dithiothreitol. The results were reported as absorbance units (ABSU).

#### Measurement of 8-hydroxydeoxyguanosin and 4-hydroxynonenal

Serum 8-hydroxy-2′-deoxyguanosine (8-OHdG) was measured with enzyme-linked immunosorbent assay (ELISA) method following the maker's instructions (Highly Sensitive 8-OHdG Check, Japan Institute for the Control of Aging, Fukuroi, Shizuoka). 4-hydroxynonenal(4-HNE) was measured with enzyme-linked immunosorbent assay (ELISA) method.

The assay variances of all methods described above were <10%.

### Other biochemical parameters

Blood glucose, triglycerides and cholesterol were determined using routine clinical chemical assays.

### Statistical analysis

Data are presented as mean±SD. All experimental data in this study were statistically analyzed with SAS 9.13. The statistical significance was evaluated using unpaired Student's t-test. Results were considered significant at *P*<0.05.
